# Editorial: The Good, The Bad, and The Ugly: Multiple Roles of Bacteria in Human Life

**DOI:** 10.3389/fmicb.2018.01702

**Published:** 2018-07-27

**Authors:** Tatiana Venkova, Chew Chieng Yeo, Manuel Espinosa

**Affiliations:** ^1^Fox Chase Cancer Center, Research & Development Alliances, Rockledge, PA, United States; ^2^Faculty of Medicine, University Sultan Zainal Abidin Medical Campus, Kuala Terengganu, Malaysia; ^3^Centro de Investigaciones Biológicas, Consejo Superior de Investigaciones Científicas, Madrid, Spain

**Keywords:** infectious diseases, antimicrobial resistance, virulence, bacterial immunity, horizontal gene spread, mobile genetic elements, bacteria and cancer

## Introduction

“*If you don't like bacteria, you are on the wrong planet.”*

(Brand, 2010).

Quoting the writer and editor Stewart Brand, summarizes the solid facts, knowledge, and fascination that we all share with regard to the smallest and simplest organisms on Earth. Bacteria are not only considered the cradle of Life, but as revealed by history and centuries of scientific interest, they are the living organisms that affect us, the Humans, most. From the moment Antonie van Leeuwenhoek observed for the first time the tiny bacterial cells under the microscope, up until the ongoing sequencing projects on the human microbiome, it has been and is an exciting journey of understanding, fighting, and using bacteria for our benefit. Many a time we tend to anthropomorphise our subjects of study, which is not necessarily a wrong practice if we remain aware of our doings and of our conclusions, thus we can artificially classify bacteria into “beneficial or pathogenic” in unequal proportion. However, with the knowledge gained throughout the years, we are still under the impression that it is still enigmatic whether we can consider bacteria as “The Good” or “The Bad” and “The Ugly” that co-habits with us. This is precisely what we have tried to do under this Research Topic with such a well-known and anthropological name, in which we have tried to combine different aspects of the bacterial world and to show how bacteria strongly influence our lifestyle. Of course, we are aware that drawing lines is a risky exercise, because what to do when a “Good” converts itself into a “Bad” and “Ugly”? Enterococci are a good example: from being a respectable member of our gut microbiome, it can turn Ugly given certain circumstances (low immuno-response on their host, we, Humans). Being scientists and trying to guide the present Research Topic, we take the scientific approach in addressing such a complex and difficult task by presenting facts and drawing conclusions that should help the readers appreciate the fascinating full spectrum of the roles that bacteria play in human life.

First and the foremost, Molecular Biology would not be where it stands today were it not for the knowledge of the basic blocks of life, i.e. DNA, RNA, and proteins, and of processes such as gene expression and its control, chromosome replication and cell division, horizontal gene transfer, cell to cell communication, DNA repair, cell immunity and cell death, that were obtained from studies in bacteria. As all these processes are relatively easy to measure in bacteria, and that the basic principles of biological regulation being same in all organisms, the knowledge gained in studying bacteria is benefiting biological sciences as a whole, including biotechnology and the emerging field of molecular medicine. In these, plasmids and phages, i.e. the bacterial mobilome, play an important role. Plasmids and phages were not only the main platform of the fundamental biological discoveries, but they are also a versatile tool for gene delivery in all organisms and the main reason for the spread of antibiotic resistance that takes a harsh toll on human life and the economy. Because many bacteria that are opportunistic pathogens live in symbiosis with plants or inhabit polluted environments, carry plasmids with genes for resistance or production of a particular enzyme, the scientific community is in a quest for finding new antibiotics to deal with infections caused by pathogenic bacteria, increase yield of plants and stimulate biodegradation. Bacterial plasmids have also been associated with and considered the “culprit” for beneficial production of animal and human food and beverages. Probiotics, which stimulate immunity and anti-inflammation, and the increasing reports of bacteria linked to cancer, are the two ultimate examples of the opposite Good and Ugly sides of bacteria. Until recently, we knew only that bacteria inhabit soil, water, extreme environments such as acidic hot springs and radioactive waste, and live in symbiotic and parasitic relationships with plants and animals. The human microbiome project opened new avenues in our understanding on the close relationship between bacteria and humans. The fact that our body, on the inside and its surface, is heavily inhabited by bacteria, urges the need for deeper investigation and we are hopeful the current Topic will provide new clues and valuable information supporting the importance of studying bacteria.

## The research topic

Despite the vast information available to date and the general belief that bacteria are more harmful than beneficial to the human population, the mere intent of proposing this Research Topic was to probe the current state of knowledge on bacteria and to figure out whether they affect our life simply in a negative/positive way, or the picture is more complex than we could have imagined. We were delighted to see that the Topic attracted the attention of 214 authors from 5 continents that responded enthusiastically with 40 original research and review articles. Our colleagues were from different scientific fields with diverse interests and points of views, which enriched enormously our understanding and knowledge on the subject. It is thus our pleasure to present the contributors to the Research Topic “The Good, The Bad and The Ugly: Multiple Roles of Bacteria in Human Life” with their invaluable reports.

## Chapters

**Chapter 1: The Beneficial Micro-World**

1.1. Source of Fundamental Biology Knowledge
1.2. Use in Biotechnology
1.3. Probiotics
1.4. Environmental Bioremediation

**Chapter 2: Pathogens unveiled**

2.1. Molecular Mechanisms
2.2. In-Depth Antibiotic Resistance
2.3. Genomics and Evolution of pathogenic bacteria
2.4. Infectious Diseases and Link to Cancer

**Chapter 3: Bacteria and Human Life**

3.1. Novel Antibiotics
3.2. Drug Delivery and Cancer Therapy
3.3. New Aspects

The Topic initiates with papers reporting on the fundamental biological discoveries that enable deeper understanding of bacterial gene expression and draw more accurate models for predicting bacterial transcription targets (Djordjevic et al.), the circuits of regulation of the replication genes in bacterial plasmids for their successful establishment in new hosts (Ruiz-Masó et al.), and a review on the possible biological roles of type II toxin-antitoxin modules, in both plasmids and chromosomes, showing evidence of the functional overlap of these modules irrespective of their genomic location (Díaz–Orejas et al.). The following two articles then deal with fundamental discoveries in the mobilome, such as the finding of two previously unknown proteins participating in the mobilization complex (relaxosome) encoded by plasmid pLS20 (Miguel-Arribas et al.), and the characterization of a mysterious protein encoded by lambda and lambdoid phages (Dydecka et al.) that may play an important role in the regulation of the decision of these phages in becoming “Ugly” (lysogeny) or “Real Bad” (lytic) in this case for the bacterial host. Two more articles relate to the Firmicutes lifestyle: how to deal with the chromosomal supercoils and the expression of genes in the pneumococcus (De La Campa et al.), one of the “Bad Ones,” usually acting as a harmless commensal in our nasopharynx, but ready to strike pneumonia when our immune system goes down, and the second dealing with the response of bacteria to stressful situations that lead to another decision: to be swept away by the stress or to survive in a dormant persister state, thus permitting the bacteria to cope with adverse (for the bacterium) situations, like facing antibiotic treatments (Moreno-del Álamo et al.).

The uses of bacteria in Biotechnology is covered by an extensive review on bacterial stationary phase promoters and their application for construction of improved gene-expression systems in recombinant protein production and in the bioremediation processes (Jaishankar and Srivastava). The “hot” topic of the CRISPR-Cas bacterial immune system that has been famously utilized in the gene-editing of mammalian cells in recent years is tackled by a closer look into its fine-tuned regulation and the proposed efficient expression of small RNAs in a narrow time interval (Rodic et al.). New insights on the genotype, enzyme production and physiological properties of beneficial bacteria such as the well-known probiotic Bifidobacteria (Alnajar et al.) and the newly-described *Pediococcus parvulus* (Pérez-Ramos et al.) are presented in depth, with a special emphasis on the role of their plasmids as in *Lactobacillus sakei* (Nácher-Vázquez et al.). Plasmids are also the main mediator of bioremediation of contaminated soils as reported by two independent groups (Garbisu et al.; Wang et al.).

The theme of the bacterial mobile genetic elements, plasmids, and phages, is extensively covered in our next Chapter (Pathogens Unveiled), as they are the driving force for horizontal gene transfer and the main cause of antibiotic resistance and virulence. We learned the interesting fact that the virulence of the pathogen *Pseudomonas syringae* is mediated by natural chimeras of distinct plasmid families (Bardaji et al.). Lean and Yeo examine our current knowledge of plasmids that are less than 10 kb in size commonly found in the nosocomial pathogen, *Acinetobacter baumannii*, in a mini-review. Some of these small plasmids harbor resistance as well as potential virulence genes whereas others are truly enigmatic. An interesting article relates the wide-spread world of prokaryotic toxin-antitoxin systems to bacterial virulence in the important pathogen of the *Campylobacter* genus, one of the “Bad Ones” because of their multiple resistances to antibiotics and their clinical relevance. Sprenger et al. show that in *Campylobacter fetus* subspecies *venerealis*, the activity of some FIC (filamentation induced by cyclic AMP) proteins resemble classical TA systems and appeared to be related to virulence. Graciela Pucciarelli et al. examine in detail the role of a disulfide bond in the major periplasmic loop of the IgaA inner membrane protein of another pathogen, *Salmonella enterica* serovar Typhimurium, in the regulation of the RcsCDB phosphorelay system, which is involved in regulating the expression of a multitude of cellular processes including motility, biofilm production and virulence. The potential use of the lectin produced by the edible snail, *Helix pomatia* agglutinin (HPA) as a novel diagnostic tool for the identification of *Streptococcus pneumoniae* is proposed by Domenech and Garcia who show that the HPA lectin specifically recognizes the terminal αGalNAc residues of the cell wall teichoic and lipoteichoic acids of *S. pneumoniae*.

The role of the bacterial viruses, the bacteriophages (or just phages), in the rapid dissemination of antibiotic resistance is presented by Valero Rello et al., whereas the entire spectrum of their impact on human health is summarized in the review article by Navarro and Muniesa. It is important to remember that bacterial phages played (and still do!) a key role in the early stages of Molecular Biology research, since they enabled the study of the control of gene expression and decision-making responses, which led to the development of controlled expression systems for protein over-expression. Further, the number of bacteriophages on planet Earth (around 10^31^) is more than any other organism, including bacteria, combined, making them a formidable evolutionary driving force.

Plasmids as vehicles for horizontal (lateral) transfer of antibiotic-resistance traits and their “evil” doings are represented by important contributions in both the Gram-positive and the Gram-negative bacterial pathogens. Identification of these genetic elements and the ways they perform their role in gene transfer are major problems nowadays, when the number of new antibacterials are dwindling. An excellent review on the replication mechanisms of several staphylococcal plasmids that mediate antimicrobial resistance is presented by Kwong et al. Águila-Arcos et al. show that in all 25 biofilm-forming clinical staphylococcal isolates that were studied, horizontal transfer and relaxase genes of two common staphylococcal resistance plasmids, pSK41 and pT181, were detected, inferring the possibility of the dissemination of antibiotic resistance to other clinical isolates. In another paper, Ares-Arroyo et al. analyze various ColE1 replicons using bioinformatics and experimental approaches. They developed a new PCR-based system for the detection and analysis of ColE1 plasmids and validated their important role in the dissemination of antibiotic resistance. Whole genome sequencing (WGS) has been routinely implemented for the identification and surveillance of *Salmonella* at Public Health England's Gastrointestinal Bacteria Reference Unit since 2014. Neuert et al. evaluated the prediction of phenotypic antimicrobial resistance in non-typhoidal *Salmonella enterica* from the genotypic profiles obtained from the whole genome sequences of 3,491 isolates received between 2014 and 2015 by Public Health England and showed that discrepancies between phenotypic and genotypic profiles were low and that by and large, WGS is suitable as a rapid means of determining antimicrobial resistance profiles for surveillance.

Looking at the other side of the coin, the study on gut microbiota and the changes in gene expression and glucose metabolism induced by antibiotic treatment shows the complex nature of our choices onto how to fight bad bacteria (Rodrigues et al.).

Taking into account the medical and environmental impact of bacterial pathogens, a special emphasis is given on understanding their genomics and evolution. *Escherichia coli* and *Bordetella*, two of the most devastating human and animals pathogens are covered extensively (Pasqua et al. and Hamidou Soumana et al.). A very important example of our change of views from “forgotten” to “Real Bad” bacteria is provided by the tuberculosis pathogen. Two papers show that *Mycobacterium tuberculosis* causing prolonged infections can acquire a limited genetic diversity, and yet there are strains prone to microevolution within the infected host (Herranz et al.; Navarro et al.). Lira et al. present comparative genomic analyses of the opportunistic pathogen, *Stenotrophomonas maltophila*, obtained from clinical as well as environmental samples and show that there are no distinct or separate clinical and environmental lineages of the pathogen. This indicates that infection is mainly due to impaired immune response of infected patients and given the biotechnological potential of *S. maltophila*, its use in its natural habitats will likely only lead to an incremental risk in acquiring infections.

Morris et al. present a comprehensive review on the role of secondary bacterial infections in increasing the morbidity and mortality of influenza infections, especially during epidemics and pandemics. The increasing antimicrobial resistance and vaccine evasion presented by these bacterial pathogens have made it even more crucial to monitor their epidemiology to better guide clinical treatment and development particularly during an influenza epidemic or pandemic. In another review, Sahan et al. show how pathogenic microorganisms can induce various levels of inflammation which can lead to DNA damage, thereby posing a risk for the development of cancers. The review focuses on *Helicobacter pylori*-mediated inflammation and gastric cancer as well as the potential role of *Fusobacterium nucleatum* in colorectal cancers besides indicating the important role of DNA repair pathways in precluding the development of such cancers.

The last Chapter (Bacteria and Human Life) starts with tackling the current shortage of effective treatment for bacterial infections and the quest for new antibiotics, being a priority of the scientific microbiological community. In a Perspective article, Grimwade and Leonard examine our current knowledge regarding the initiation stage of bacterial chromosomal replication, mediated by the bacterial orisome, and they identify potential targets that could prevent bacterial chromosomal replication, which therefore could serve as targets for novel antibacterial compounds.

Molecules that are able to inhibit conjugation (COINS) have been proposed and thought to provide a novel avenue to combat the spread of antibiotic-resistance traits encoded by mobile elements. Some effective COINS were discovered a few years ago, and the strategies to identify and to further develop them are reviewed by Cabezón et al. Another approach to deal with pathogens is related to the hindering of their lifestyle. Many bacteria (and the Bad Ones are no exception) grow happier when they grow together forming biofilms that will stick to living (teeth, nasopharynx) or implant (catheters, prosthesis) surfaces to better communicate among them as well as to colonize new niches. Inhibitors of biofilm formation are an interesting source of potential antimicrobial drugs that is recorded by Vaishampayan et al.

One of the major difficulties in the drug discovery field is how to deliver the desired drug so that it reaches its final target. Drug delivery strategies can be costly and time-consuming: this could make a cleverly designed drug unable to be used because of the lack of proper delivery procedures. Exploring this field has been the subject of the Llosa's laboratory for years. They provide now an insightful review on how to use the bacterial Type IV secretion system pathways to deliver and to stably integrate into mammalian cell chromosomes with desired traits that would, in time, lead to anti-tumor drugs (Guzmán-Herrador et al.).

The use of several bacterial species in cancer therapy is examined in a Perspective article by Gabriela Kramer et al., thus contrasting with the “Bad” side of other bacterial species that have been shown to be potential causal agents for cancer (Sahan et al.). Intriguingly, attenuated mutants of the pathogen, *Salmonella enterica* serovar Typhimurium have been shown to invade and destroy a broad range of cancer cell types *in vitro* and are so far, the most efficient anti-tumor bacteria in experimental models of cancer.

Finally, unexpected facts on bacteria are unveiled by the last 2 articles in our Topic. Molina-García et al. show that bacteria can be the ideal model for studying human neurodegenerative diseases, whereas Javan et al. report on the bacterial thanatomicrobiome that could aid in forensic investigations.

## What is next?

Despite the vast knowledge on bacteria, including the current Research Topic, new and exciting scientific reports are coming up every day. Among those, the human microbiome project plays a central role on revealing the true interaction between us, the humans, and those “primitive” but powerful living organisms that have now been shown to play central roles in shaping our health and our environment. As the collection of articles in this Research Topic has shown, bacteria do indeed display all facets of the “Good,” the “Bad,” and the “Ugly,” and like everything else in this world of ours, all three facets co-exist as a dynamic, chaotic whole.

## Author contributions

All authors listed have made a substantial, direct and intellectual contribution to the work, and approved it for publication.

### Conflict of interest statement

The authors declare that the research was conducted in the absence of any commercial or financial relationships that could be construed as a potential conflict of interest.
